# Breaching the Fortress of Tumor Microenvironment to Control Cancer Metastasis

**DOI:** 10.3390/cancers15184562

**Published:** 2023-09-14

**Authors:** Aayami Jaguri, Aamir Ahmad

**Affiliations:** 1 Weill Cornell Medicine-Qatar, Doha 24811, Qatar; aaj4005@qatar-med.cornell.edu; 2 Translational Research Institute, Academic Health System, Hamad Medical Corporation, Doha 3050, Qatar; 3 Dermatology Institute, Academic Health System, Hamad Medical Corporation, Doha 3050, Qatar; 4 Department of Dermatology and Venereology, Rumailah Hospital, Hamad Medical Corporation, Doha 3050, Qatar; 5 Department of Bioengineering, Integral University, Lucknow 226026, UP, India

As the primary cause of death for >90% of cancers, metastasis is the fourth and final stage of cancer during which cells gain the ability to leave their primary site, invade surrounding tissues, and disseminate to distant organs [1]. Research over the last two decades has been instrumental in revolutionizing our understanding of cancer progression and metastasis. While tumorigenesis was previously thought to have a predominantly genetic basis, recent studies have shown that the cellular and structural components of the tumor microenvironment (TME)—initially believed to be bystanders in tumorigenesis [2]—serve an equally important function [3,4]. The TME constitutes of the extracellular matrix (ECM), the basement membrane (BM), tumor cells, immune cells, endothelial cells, pericytes, and signaling molecules involved in the regulation of tumor progression [5,6]. Depending on the primary site of the tumor, the characteristics of cancer cells, the traits of patients, and the stage of the tumor, the cellular and structural features of the TME can vary and can either help support or suppress tumors.

In the review entitled “The Role of Tumor Microenvironment in Cancer Metastasis: Molecular Mechanisms and Therapeutic Opportunities”, published in the 13th volume of *Cancers* in 2021, Neophytou and co-authors address the roles of various cellular and structural TME components in the invasion-metastatic cascade; TME-targeting strategies to inhibit tumorigenesis; and approaches to reorganizing the TME and improving therapeutic efficacy during metastasis [7]. 

As pointed out by Neophytou et al. [7] in their article, immune cells constitute an important part of the TME, owing largely to their crucial functions within the TME through their interactions with, and actions on, cancer cells at various stages of metastatic progression [8]. In particular, CD8+ T cells and natural killer (NK) cells play pivotal roles in helping restrict metastasis, and when they are depleted or dysfunctional in tumors, it leads to increased metastasis [9,10]. Tumor-associated macrophages (TAMs) can have both tumor-supporting and tumor-suppressing functions, depending on their subtype [11]. M1-type TAMs suppress tumors through phagocytosis or by inducing immune responses targeting cancer cells. On the other hand, M2-type TAMs exert tumor-supporting effects, which include promoting metastasis, immunosuppression, angiogenesis, and anticancer drug resistance [11,12]. Mesenchymal stem cells (MSCs) have been shown to promote the proliferation and migration of cancer cells through the secretion of exosomes carrying miRNAs [13,14]. Cancer-associated fibroblasts (CAFs) secrete signaling molecules that promote cancer cell survival, in addition to reorganizing the ECM and creating tracks for cancer cells to directionally migrate [15,16].

Additionally, the ECM plays several determinant roles in tumorigenesis and metastasis. In tumorigenesis, the ECM serves as a biological barrier preventing the dissemination of tumor cells from the primary site to distant organs. During tumor progression, however, the ECM is remodeled, creating metastasis-promoting conditions [17]. This remodeling of the ECM is characterized by excess collagen synthesis which leads to fibrosis. Fibrosis increases tissue stiffness and mechanical compression of tumor cells, which often facilitates the migration of cancer cells [18]. Tumor stiffening also induces hypoxia, which stimulates angiogenesis, thus increasing the supply of oxygen and nutrients to the tumor, in addition to inducing numerous signaling cascades which promote proliferation, migration, and metastasis.

At various stages of metastatic progression, the authors [7] describe how cellular components of the TME can be targeted by pharmaceuticals to combat metastasis. Targeting TAMs at the primary site of the tumor is one encouraging approach. TAMs are known to facilitate epithelial-to-mesenchymal transition (EMT) in cancer [19], a process whereby epithelial cells convert to a mesenchymal phenotype and can therefore migrate out of the primary site and disseminate to other organs. This step in the TME can be targeted by JWH-015, a cannabinoid receptor 2 (CB2) agonist which inhibits EMT [20] and downregulates the expression of matrix metalloproteinases (MMPs) secreted by TAMs and other TME cells. MMP activity, a major driver for angiogenesis, has additionally been shown to be inhibited by several compounds, including bisphosphonates, carbamoylphosphonates, and thiols [21,22,23]. Clinical studies, however, have found these approaches to have limited efficacy [24,25,26]. Moreover, dose-limiting side-effects of MMP inhibitors have posed a difficulty—many studies indicate that the dosages used in clinical trials are insufficient to inhibit MMP expression [27,28,29]. Colony-stimulating factor-1 (CSF-1/M-CSF), which is pivotal in the recruitment of TAMs to the tumor site, is another promising cellular target; pharmaceuticals inhibiting the interaction of CSF and the CSF-R1 receptor can help block metastasis [30]. Furthermore, exosomes loaded with taxol, or miR-124 and miR-145 mimics, have been reported to significantly reduce the migration of tumor cells and the growth of metastatic tumors [7,31]. The blood microenvironment can also be targeted through the inhibition of platelet function, or through the modulation of cytokine content, which leads to the blocking of metastasis. A number of drugs have been researched for their anti-platelet activity, including APT102, an ADPase that restricts platelet activity, and the BMP22 ATX inhibitor, which reduces metastasis through the inhibition of the LPA/ATX signaling axis [32,33].

Physiological aberrations in the TME can be countered through vascular remodeling and stroma normalization. The vascular remodeling strategy seeks to re-establish normal vasculature through restoring the normal balance between pro- and anti-angiogenic signaling. One important target for this approach is VEGF signaling, which can be regulated through the drug Bevacizumab that prevents the binding of VEGF to its receptor [34]. Anti-angiogenic peptides—endostatin, growth factors, and chemokines, for example—have also demonstrated high success rates for vascular remodeling in clinical studies [7]. On the other hand, the stroma normalization strategy aims to restore normal vessel function by reducing the compression of intratumoral vessels and tumor stiffening that leads to metastasis [35,36]. This can be achieved via CAF reprogramming and ECM remodeling. CAFs take part in ECM deposition, which increases tissue stiffness, in addition to being involved in inflammatory signaling. Targeting CAFs has been reported to decrease tissue stiffness and interstitial fluid pressure in tumors, as well as improve the activity of anticancer agents such as taxol and 5′-fluorouracil [37]. Moreover, combined targeting of the immunosuppressive ligand TGF-β secreted by CAFs and the PD-1/PD-L1 axis has shown significant anti-tumor effects [38].

As novel therapeutic approaches to metastasis continue to be developed, Neophytou et al. highlight the need to take into account the variation between the TME at the primary and secondary sites of the tumor. A study by Cacho-Diaz et al. notes that the tissue in which the tumor originated at the primary site impacts the type of tumor that develops at the secondary site, as well as the metastatic outgrowth [39]. These findings suggest that while regulating one component of the TME at a time may have limited clinical efficacy, strategies targeting multiple TME components are likely to demonstrate greater success in combating metastasis. As research on the development of combination therapies for cancer continues to grow, taking into account the characteristics of TME components is an important step to optimize drug delivery and the therapeutic response.
